# Achromatic beam deflector with electrodynamic phased arrays

**DOI:** 10.1038/s41377-025-01936-5

**Published:** 2025-08-18

**Authors:** Jungkwuen An, Young Kim, Yunhee Kim, Hoon Song, Chunghwan Jung, Kanghee Won, Junsuk Rho, Hong-Seok Lee

**Affiliations:** 1 Visual Technology Team, Samsung Research, Seoul, 06765 Republic of Korea; 2 Advanced Sensor Lab, Device Research Center, Samsung Advanced Institute of Technology, Suwon, 16678 Republic of Korea; 3 Department of Chemical Engineering, Pohang University of Science and Technology (POSTECH), Pohang, 37673 Republic of Korea; 4 Department of Information Display, Kyung Hee University, Seoul, 02447 Republic of Korea; 5 Department of Mechanical Engineering, Pohang University of Science and Technology (POSTECH), Pohang, 37673 Republic of Korea; 6 Department of Electrical Engineering, Pohang University of Science and Technology (POSTECH), Pohang, 37673 Republic of Korea; 7 POSCO-POSTECH-RIST Convergence Research Center for Flat Optics and Metaphotonics, Pohang, 37673 Republic of Korea; 8 Major of Electrical Engineering, College of Engineering, Pukyong National University, Busan, 48513 Republic of Korea

**Keywords:** Displays, Metamaterials, Nanophotonics and plasmonics, Micro-optics, Imaging and sensing

## Abstract

Since flat optics has the feature to implement a compact system, they are widely used in various applications to replace bulky refractive optics. However, they suffer from chromatic aberrations due to dispersion, limiting their effectiveness to a narrow wavelength range. Consequently, diffractive optics has been applied for dynamic beam steering within a specific wavelength region or for static steering across multiple wavelengths. This limitation has made it challenging to implement dynamic beam steering in full-color display applications. To address this issue, we developed a multi-wavelength-based optical architecture that mitigates chromatic aberrations. This system incorporates color-selective retarders, half-wave plates, polarization plates, and beam deflectors. We experimentally demonstrated an achromatic beam deflector using a dynamic phase array in transmission mode, achieving continuous tunable beam steering over multiple wavelengths at 460, 520, and 638 nm.

## Abstract (web summary)

Achromatic beam deflector eliminates chromatic aberrations in dynamic beam steering using color-selective retarders and liquid crystal phase arrays, enabling independent control of RGB wavelengths for display applications.

## Graphical abstract

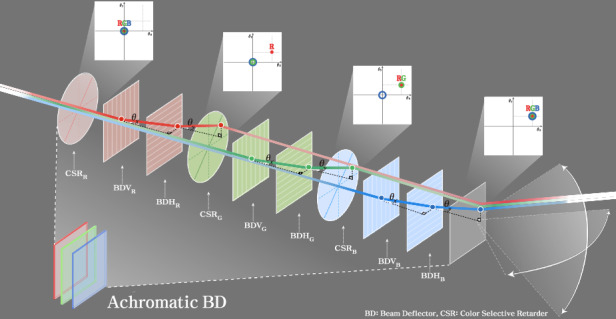

## Introduction

Optical beam steering is essential for applications that require precise control of light propagation and various approaches to developing optical systems have been investigated such as free-space optical communications^1–3^, light detection and ranging^4–7^, microscopies^8–10^, displays^11–14^, and virtual/augmented reality headsets^15–17^. The approaches for optical beam steering can be broadly classified into two major groups: mechanical and nonmechanical.

Classical mechanical beam steering methods have attracted substantial research interest in the field of microelectromechanical systems (MEMS) over the years. Various approaches have been explored, including rotating prisms^18–20^, mirrors^21–25^, piezoelectric actuators^26–28^, and electro-wetting^29–31^, highlighting diverse strategies for achieving precise optical beam control. Optical beam steering devices based on MEMS technology provide opportunities for integration into contemporary optical systems and serve as effective solutions for a range of optical applications. However, size constraints introduce several challenges, including limited deflection angles, vibration sensitivity, and fabrication difficulties.

To overcome these challenges, numerous nonmechanical beam steering technologies have emerged, which control the propagation of light through refractive, diffractive, or wavefront modulation mechanisms. The primary advantage of these approaches lies in their inherent robustness, achieved by eliminating moving parts, unlike mechanical beam steering techniques that require moving parts to redirect light. Nonmechanical beam steering methods mainly involve acousto-optic^32–34^, electro-optic deflectors^35–37^, liquid crystal (LC) technologies^38–42^, metasurfaces^43–50^, to name a few. Among them, LC-based beam steerers are highly competitive in this field due to their high birefringence, which allows for efficient light steering by generating precise phase distributions for a given polarization, all with low power consumption^51^. LC-based nanophotonic devices have been developed to facilitate applications involving variable period^52,53^, variable blazed^54–57^, and diffractive lenses^58–60^. Although diffractive optics is advantageous due to their compactness and thinness, they inevitably produce chromatic aberration as the diffraction angle varies with wavelength. As a result, the steered light exhibits varying angles for each of the red, green, and blue (RGB) colors when these wavelengths are incident on the grating surface^61–64^. Various studies have been developed to correct chromatic aberrations inherent to diffractive optics over multi-wavelengths, including a new method of light manipulation based on scattering from metasurface structure^65–71^. However, a structure capable of dynamically steering light while simultaneously responding to multi-wavelengths within the visible spectrum has not been developed yet. In this paper, we propose an electro-optic beam steering device without chromatic aberration for incident light in the visible spectrum.

## Results

### Chromatic aberration in the beam deflector and its compensation

Beam steering with LC leverages its anisotropic characteristics to modulate the phase of light as it travels through a specified path, enabling precise control over the accumulated phase. This is commonly implemented using a matrix of LC pixels, each inducing a local phase shift between 0 and 2π, which controls the phase front to direct the beam in a chosen direction through reflection or transmission mode. Despite the benefits of the reflection mode, such as accommodating a broader range of materials and achieving strong phase shifts over shorter paths, we chose to fabricate a beam deflector (BD) of transmission type using indium tin oxide (ITO) electrodes on glass substrates. This approach allows for easier alignment of light sources and detectors in a linear setup, making it more suitable for integration into compact systems.

**Fig. 1:**
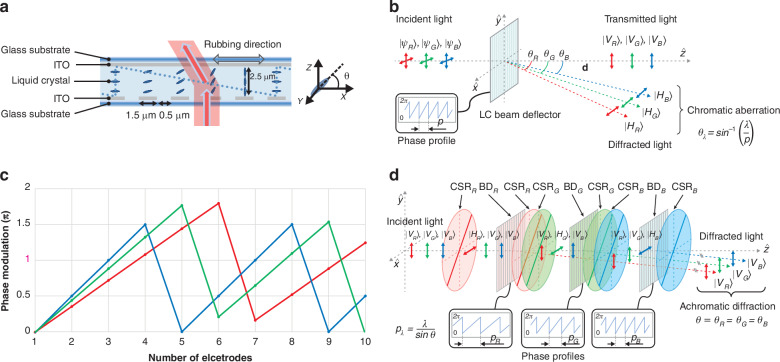
**Principle and design of the beam deflector (BD) device with polarization-dependent phase modulation**. **a** Schematic of the BD device. The upper glass substrate is coated with a planar indium tin oxide (ITO) layer, while the lower glass substrate is coated with strip ITO electrodes along with the *y*-axis. The LC directors are initially aligned homogeneously along the rubbing direction, the *x*-axis, which is perpendicular to the strip electrodes. **b** Selective response with the linear polarization direction of polarization dependence of BD and chromatic aberration depending on the difference in wavelength. **c** Different phase profiles of 2π phase modulation for RGB wavelengths. **d** Concept of achromatic beam deflector (ABD) by exploiting the polarization state of light. Color-selective retarder (CSR)_R, G, and B_: CSR for red, green, and blue, BD_R, G, and B_: BD for red, green, and blue

The BD is composed of ITO electrodes configured in a one-dimensional (1D) architecture, which is illustrated in Fig. 1a. The surface facing the nematic LC layer of the upper glass substrate is coated with a planar ITO layer, working as the grounding electrode. On the other side, the surface facing the nematic LC layer of the lower glass substrate is coated with strip ITO electrodes, acting as the driving electrode. The strip electrodes on the lower substrate are in the direction of the *y*-axis, and the inner surfaces of both substrates are coated with polyimide films and rubbed in the *x*-axis. The optical axis of the LC is initially aligned perpendicular to the strip electrode pattern with a small pretilt angle in the absence of an applied voltage. The BD is driven by applying 9 V to the grounded upper planar ITO electrode and 0–18 V to the lower strip ITO electrodes.

In the voltage-on state, the voltage across the strip ITO electrodes and the planar ITO electrode generates spatially nonuniform electric fields, and the LC directors are then reorientated by them. The local tilt angle *θ* of the LC directors increases with stronger electric fields between the upper and lower electrodes, indicating that *θ* is minimized when the potential difference between the upper and lower substrates is low and maximized when the potential difference is high. When a linearly polarized light beam is incident parallel to the rubbing direction, the effective refractive index experiences^72^

1 $${n}_{{eff}}\left(\theta \right)={{n}_{o}{n}_{e}/({{n}_{o}}^{2}{\sin }^{2}\theta +{{n}_{e}}^{2}{\cos }^{2}\theta )}^{1/2}$$

where ${n}_{e}$ and ${n}_{o}$ are the extraordinary and ordinary refractive indices of the LC material, respectively.

By calculating the final effective refractive index (*n*_*eff*_) distribution, the difference of phase retardation of BD is determined. The accumulated phase retardation *φ*, resulting from the maximum difference between the refractive indices of the incident light beam, is calculated by^73,74^

2 $$\Delta \varphi =\frac{2\pi \cdot \Delta n\cdot d\,}{\lambda },\Delta {{n}}={{{n}}}_{{{e}}}-{{{n}}}_{{{o}}}$$

where *λ* is the wavelength of the light, *d* is the thickness of the LC layer, and birefringence Δ*n* is defined as the difference between the extraordinary (*n*_*e*_) and ordinary (*n*_*o*_) refractive indices.

To achieve a minimum of 2π modulation across all wavelengths, the thickness of the LC layer was determined by the longest wavelength, red, as the reference. The linear phase profile assigned to the BD should be wrapped by $2{\rm{\pi }}$ phase, because the dynamic range of the optical path length difference induced by the LC is limited by its thickness. Although various shapes of optical prisms can be achieved through phase retardation equation as shown above, we implemented a sawtooth phase profile to steer light in a single direction through BD, making it suitable for display applications.

In order to distinguish between (H)-type and (V)-type BD, (H)-type BD is defined as having a vertical electrode pattern to deflect light horizontally (i.e., in the left-right direction), whereas (V)-type BD is defined as having a horizontal electrode pattern to deflect light vertically (i.e., up and down). Phase retardation occurs when the optical axis of the LC is parallel to the direction of linear polarization, the polarization state parallel to the director of the LC can be deflected by the (H)-type BD whereas the orthogonal polarization transmits the (H)-type BD without deflection as shown in Fig. 1b.

The diffraction angle, ${\theta }_{\lambda }$ is determined by both the wavelength of light, $\lambda$ and the period of the diffraction medium, $p$ as following. ${\theta }_{\lambda }={\sin }^{-1}\left(\frac{\lambda }{p}\right)$. As described in Eq. (3), the steering angle is determined by the channel number of the prism and the cell size. Steering angle *θ*_*str*_ can be derived as follows:

3 $${\theta }_{{str}}={\sin }^{-1}\frac{\lambda }{n\cdot p}\,,n=\frac{m}{i}(0 < {{i}}\le 360)$$

where *λ* is the wavelength of the light, *n* is the total channel number for one unit prism that is defined by *m*/*i*, *m* is the total number of channels, and *i* is the total number of unit prisms. The electrode pitch *p* is defined as 2 μm, consisting of an electrode width of 1.5 μm and an inter-electrode spacing of 0.5 μm. In Eq. (3), the diffraction angle is proportional to the wavelength, and the maximum steering angles are calculated as 6.60°, 7.72°, and 9.17° for 460 nm, 520 nm, and 638 nm, respectively. From the viewpoint of the whole device, considering all three wavelengths, the maximum diffraction angle should be determined by blue, which has the shortest wavelength among them.

Since BD has the common period $p$, the diffraction angle differs for each wavelength, which causes the chromatic aberration of a single BD. The structure of a BD using 2${\rm{\pi }}$ phase wrapping distorted phase distributions due to the phase discontinuity is described in Fig. 1c. The beam steering device utilized in this study features ITO electrodes with a 2 μm pitch and generates blazed phase profiles to electrically steer light in a desired direction. To direct light in a single direction when the three RGB wavelengths are incident simultaneously, the optical phase prism must be designed to optimize the wavelength-dependent diffraction angles. For instance, to direct all RGB light at an angle of 3.29°, the optical phase profile must repeat every 11.09 μm for red, 9.04 μm for green, and 8 μm for blue. For a BD with a 2 μm pitch electrode, a 2π modulation unit prism should be generated using 5.55, 4.52, and 4 electrodes for RGB, respectively. However, as the electrode can only represent integers and not decimals, phase discontinuities arise. The 2π modulation blazed phase profile is repeated several times across 720 channels, enabling the creation of a prism compatible with each RGB wavelength by fine-tuning the start and end values of each unit prism using an optimized algorithm.

The structure that selectively diffracts light of three wavelengths of RGB was described previously, and compensation for chromatic aberration will be explained as follows. Figure 1d shows the schematic for the 1D ABD system with three dynamic phased arrays. Each of the three BD is configured to deflect only the target wavelength out of the three incident lights, depending on their polarization. The color-selective retarders (CSRs) are required to control the polarization states independently among three wavelengths. It works as a half-wave retarder with 45° fast optic axis for designed wavelength while the other wavelengths undergo no retardation (or multiple of full-wave retardation). Thus, CSR only switches the polarization state of the target wavelength between two orthogonal polarization states. By switching between the orthogonal polarization states, RGB light can be controlled independently to arbitrary angles. It includes the case for the same diffraction angle for three different colors, which enables ABD. For the target wavelength, which is distinguished by the polarization state, the BD sets the appropriate phase profile with period of ${p}_{\lambda }=\frac{\lambda }{\sin \theta }$ where $\lambda$ is the target wavelength and $\theta$ is the diffraction angle. The 1D ABD beam steering can be used in a promising platform to achieve arbitrary 1D beam shaping at high speed, continuously tunable true-time delay lines in phased array antennas, a light focus in 1D to act as a cylindrical lens, and waveguide-integrated devices^75–79^.

### 2D achromatic beam deflector

**Fig. 2:**
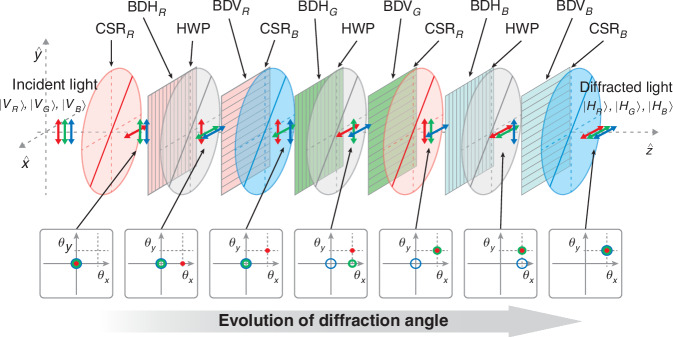
**Structure of two-dimensional ABD**. CSR _R and B_: CSR for red and blue wavelength, BDH _R, G, and B_: (H)-type BD for red, green, and blue, BDV _R, G, and B_: (V)-type BDs for red, green, and blue wavelength. Six BDs are used to control three colors in two dimensions. Note that there are no CSRs for green wavelength, since the required polarization state after each deflection differs for each step due to the arrangement of (H)-type and (V)-type BDs

**Fig. 3:**
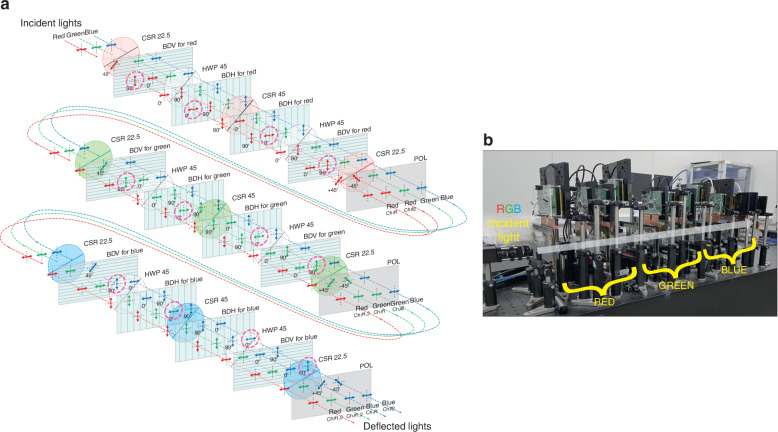
**Structure of the dual channel 2D ABD**. **a** Three BD stages are placed in sequence for each red, green, and blue wavelength. The deflected light components are highlighted by the pink dashed circles after each BD. **b** Actual experimental setup of the ABD with dynamic phased arrays and the prototype includes optical components (CSR, HWP, Polarization plates), BDs and control boards, power connectors, and R, G, B laser sources

For complete two-dimensional (2D) control of RGB light, additional beam steering is required in the vertical direction as well as the existing horizontal direction, which demands the use of additional (V)-type BDs. 2D beam steering is effectively realized by integrating two orthogonally oriented 1D BDs with intersecting electrode configurations. For optimal efficiency, the BD requires the input beam to be linearly polarized along the initial alignment of the LC. Thus, a 90° polarization rotator must be placed between the (H)-type and (V)-type BDs, and the (V)-type BDs are followed by the half-wave plate (HWP) after each (H)-type BD as shown in Fig. 2. It can be further extended the degree of freedom (DoF) so that one can construct the dual channel of 2D ABD, which independently controls two beams of RGB light, simultaneously. This can be achieved by launching the initial polarization state as the superposition of two orthogonal states |*V*〉+*H*〉| as shown in Fig. 3.

Figure 3 shows each layer of the unit BD to explain how each unit BD selectively deflects color among the overall structure of the 2D ABD with dual channels described above. The unit BD consists of four BDs for the channel #1/#2 and (H)-/(V)-type, three CSRs for 22.5° and 45°, and two HWPs. Also, each of the two (H)-type and (V)-type BD is arranged to correspond to the channel #1 and #2, respectively.

Initially, the beam is incident on the first CSR with a 22.5° optical axis with horizontal polarization using a linear polarizer. When the optical axis passes through 22.5° CSR for red, the polarization of the red wavelength is rotated by 45°, and the rest of the green and blue wavelengths remain horizontal without changing the polarization. For red beam polarized at 45°, its vertical (90°) component is used as a beam directed to the channel #1, while the horizontal (0°) component is used to the channel #2 as they pass through the following 4 BDs. The vertical (90°) component of the channel #1 beam reacts with the LC in the first (V)-type BD and is vertically deflected. Before entering the second (H)-type BD, the polarization of all beams rotates by 90°, passing through the HWP with a 45° optical axis. Then, the red beam of channel #1 is deflected horizontally in the second (H)-type BD. The CSR for red with a 45° optical axis switches the polarization of the red wavelength between horizontal and vertical polarization. In this case, the previously controlled red beam for the channel #1 has the same vertical polarization state as the rest of the green and blue beams, and only the red beam for the channel #2 has horizontal polarization which is perpendicular to the polarization of remaining 5 beams. The third (H)-type BD deflects only the red beam for the channel #2. Next, the polarization of the entire beam is rotated by 90° with the HWP having an optical axis of 45°, and then the vertically polarized light is incident on the fourth (V)-type BD. At this time, the red beam of the channel #2 is deflected in the vertical direction, and control of both beams of the red wavelength is terminated. Then, the 22.5° CSR was used to make the polarization state of the red beams of both channels to ±45° so that their power remains the same as they pass through the final polarizer.

The overall process for the unit BD stage can be expressed as the product of Jones matrices for each optical element as below

$$J={J}_{{POL}}\cdot {J}_{{CSR}22.5}\cdot {J}_{{BDV}}\cdot {J}_{{HWP}45}\cdot {J}_{{BDH}}\cdot {J}_{{CSR}45}\cdot {J}_{{BDH}}\cdot {J}_{{HWP}45}\cdot {J}_{{BDV}}\cdot {J}_{{CSR}22.5}$$

$$J=\frac{1}{2}\left[\begin{array}{cc}1 & 0\\ 0 & 1\end{array}\right]{{{for}}\; {{target}}\; {{wavelength}}}$$

$$J=\left[\begin{array}{cc}1 & 0\\ 0 & 1\end{array}\right]{{{for}}\; {{non}}-{{target}}\; {{wavelength}}}$$

The details of polarization evolution among optical elements in the achromatic BD using Jones formalism are available in Supplementary Note 1. The beams passing through the unit BD for red are then incident on the unit BD for green and blue consecutively, which are composed in the same way as that of red.

The intensity loss of each optical component was measured, and the unit BD for red was measured to have an efficiency of ~31% relative to the incident light (Supplementary Note 2). It is attributed to depolarization occurring within the LC layer, the retardation films, and reflections at the glass surface. Depolarization can partially result from phase differences between polarization components caused by the misalignment of LC molecules due to irregularities in the rubbing process. Additionally, it can also be induced by light scattering from spacer balls used to maintain the uniform thickness of the LC cell. This can be accomplished by minimizing alignment defects through methods such as precise rubbing techniques or photoalignment, utilizing high-purity LC materials, and employing optimized anchoring layers to mitigate depolarization effects. Additionally, applying anti-reflection coatings and index-matching layers can help reduce multiple reflections and the associated polarization changes.

Table 1 shows the sequence of the entire operation until the light of three wavelengths of RGB passes through each component and finally exits. 2D ABD with dual channels consists of a total of 12 individual BDs, which control each of the two pairs of RGB beams in desired directions by deflecting them vertically/horizontally. The key to this structure is that while the target beam assigned to the target individual BD is deflected, the remaining 5 beams must maintain orthogonal polarization with respect to the target beam so that they are not deflected by the corresponding BD. For this reason, not only a HWP that simultaneously rotates polarized light in the entire wavelength band, but also CSRs with different retardation for each wavelength are required. The described structure is also compatible with circularly polarized and structured light. To enable this functionality, a quarter-wave plate (QWP) is positioned at the input to convert circular polarization into linear polarization. At the output, another QWP is added to transform the resulting linear polarization back into circular polarization. Also, structured light can be controlled in the same manner, provided its polarization is spatially uniform, whether it is linearly or circularly polarized.

**Table 1:**
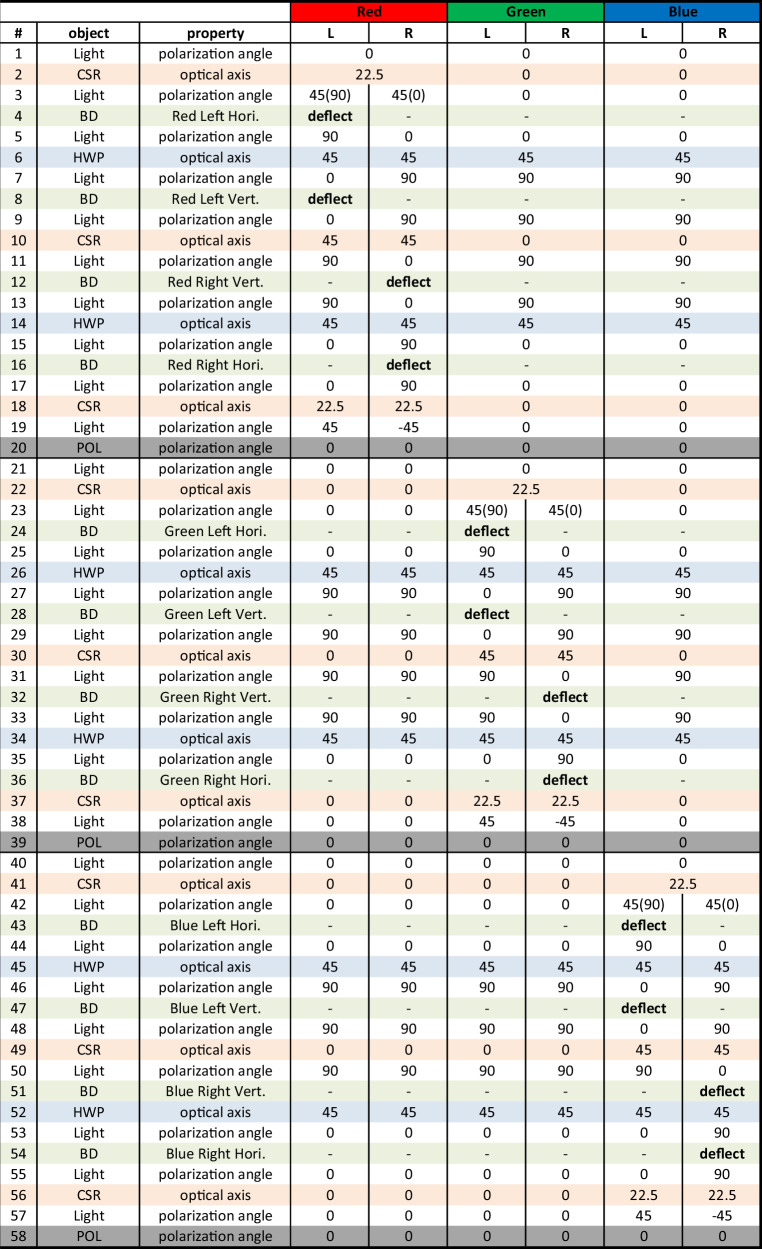
Structure of 2D ABD with dual channels for RGB wavelengths

## Discussion

**Fig. 4:**
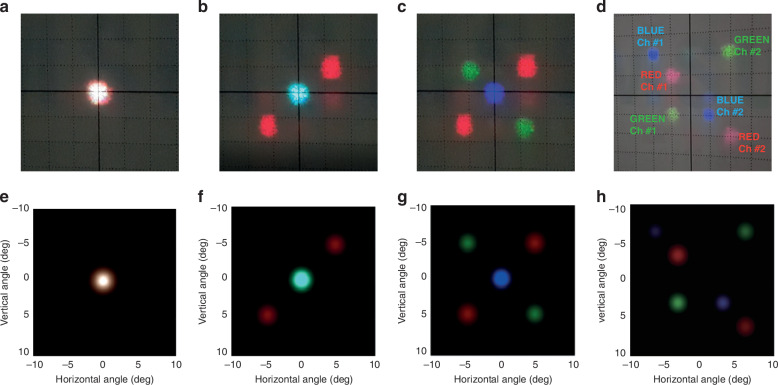
**Demonstration of R, G, B incident light using dynamic phased arrays of 2D ABD with dual channels**. **a** The simultaneous incidence of 3 light sources—R, G, and B—passing through 12 BDs in the absence of an applied voltage. **b** Only red incident light is deflected into two channels, while green and blue incident lights still remain in DC without deflecting. **c** Only green incident light is deflected into two channels following the previously deflected red incident light. The blue incident light does not deflect and still remains in DC. **d** Blue light is deflected into two channels. **e**–**h** Fourier optic simulation results of experiments correspond to (**a**–**d**). The movie clip is available in Supplementary Movie 1, where an angular resolution as small as 0.021° is demonstrated

Figure 4 shows that the beams of the RGB wavelengths are independently deflected for dual channels. Three color laser diodes are used as coherent light sources and they are summed as they pass through this setup shown in Fig. 3b. Figure 4b shows the results of the outcome of steering the red light source in different directions using two sets of (V)-type and (H)-type BD_R_, while the green and blue incident lights stay in the DC state without experiencing deflection. When voltage is applied to the two sets of (V)-type and (H)-type BD_G_ responsive to green light, additional deflection takes place in two directions, while the previously deflected red light and the unaffected blue light remain unchanged as shown in Fig. 4c. Finally, it can be seen that the blue light source is steered in two distinct directions, and the RGB wavelengths are independently deflected in separate directions. This demonstrates that when RGB light is simultaneously incident, the BD_R_, BD_G_, and BD_B_ components can be dynamically controlled in real time to steer in the desired direction for each of the two channels.

The proposed 2D ABD with dual channels can be used to increase efficiency in holographic image expression in flat panel displays. An and Won et al. have demonstrated an architecture that combines the advantages of both BDs and beam expanders (BEs), enabling the path towards slim-panel holographic video display^14^. The waveguide-based BE is designed to increase the field of view by transforming a narrow and collimated input beam into a broader output beam^80^. A distinct advantage of this approach lies in its ability to achieve étendue expansion through small entrance area replication, effectively overcoming the limitations that other architectures face due to étendue constraints^81^. Because the BE guides light along a predetermined path through the total internal reflection mechanism, the input grating must be designed according to the specific angle of the incident light.

**Fig. 5:**
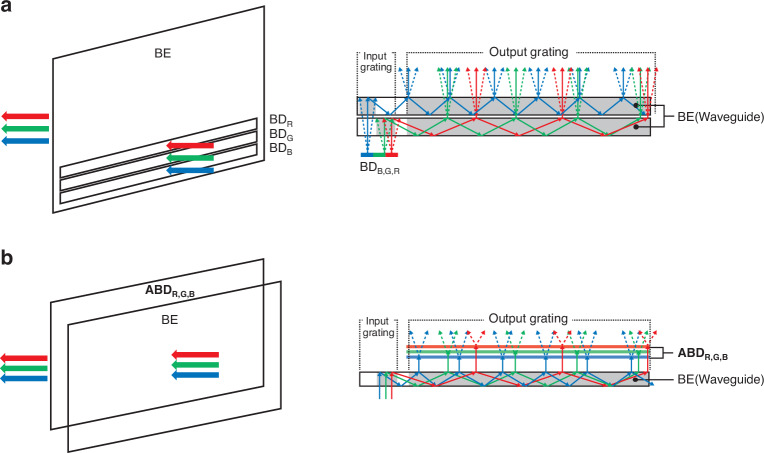
**Two different system configurations depending on the ABD architecture application**. **a** Schematic of three light sources incident on each BD for red, green, blue, and then combined within a beam expander (BE) without ABD architecture. Light with various angles, deflected by the BD, is incident on the input grating of BE. **b** Schematic of three light sources incident on BE at a certain angle and then deflected in the BD set with ABD architecture

To implement a holographic display without 2D ABD with dual channels, three deflected light sources from each BD had to be incident on the BE separately and then the three incident lights are combined at the output grating as shown in Fig. 5a. This is because the input grating of BE must be designed for all angles deflected from the BD, so the efficiency of the waveguide is inevitably reduced. However, if three incident lights can be individually controlled by the use of proposed ABD architecture, the BD can be placed after the BE as shown in Fig. 5b. This enables the input grating of the BE to be designed and be optimized at a single incident angle, so the efficiency of the waveguide can be greatly improved.

In conclusion, we have demonstrated a dynamic ABD architecture for three wavelengths. In particular, the proposed ABD architecture consists of transparent types of BDs, and it has the advantage of being able to implement a slim form factor system. The current proof-of-concept setup is relatively large; however, optical components such as HWPs and polarizers can be implemented as thin films, and the BD and CSR can be combined into a single unit with a BD cell. This approach not only reduces the overall size but also eliminates Fresnel losses at each layer, thereby enhancing transmission efficiency. Additionally, we designed the system to maximize the DoF with multiple BDs (12 DoF: R/G/B × H/V × R/L). However, by applying sinusoidal waves, the two BDs used to project to the left and right eyes can serve both eyes, effectively reducing the current 12 to 6 BDs.

We describe a framework for overcoming the limitations of chromatic aberration and have developed an approach that enables operation in three different wavelengths of 460, 520, and 638 nm by the use of CSRs as a key component. Our experimental results validate this approach, and the proposed novel architecture is expected to be widely used in various industries such as display areas in holographic display and virtual/augmented reality displays, applications spanning a broad range of wavelengths, such as those in sensors and telecommunications (Supplementary Note 4).

## Materials and methods

### Fabrication techniques

ITO-coated glass substrates were sequentially cleaned using deionized water, acetone, and ethanol, followed by drying and UV ozone treatment for 30 min. Polyimide (PI) was coated using a spin coater (MIDAS-5000A). A three-step spin process was employed to achieve uniform film thickness: a first low-speed spin at 1500 rpm for 30 s to ensure uniform spreading, followed by a high-speed spin at 3000 rpm for 60 s to attain the target thickness and enhance uniformity, and a final low-speed spin at 1500 rpm for 30 s for stabilization. This process resulted in a uniform PI layer with a thickness of ~100 nm. To remove residual solvents and eliminate tackiness from the PI film, a soft bake was conducted on a hot plate (WH210) at 120 °C for 5 min. Subsequently, a hard bake was performed to induce polymer imidization and form the alignment layer, using a hot air furnace (Memmert-UF55) at 280 °C for 60 min. For ECB (Electronically Controlled Birefringence) mode LC alignment, mechanical rubbing was carried out using a physical rubbing machine (SHINDO-RUBBING M/C), aligning both upper and lower substrates in a parallel direction. The circular glass substrates were then cut to the desired size using a scribing system (SHINDO-SCRIBE). To maintain a uniform cell gap, spacers with a diameter of ~2.5 μm were dispersed onto the lower substrate using a spacer sprayer (SHINDO-SPACER SPRAYER). Following this, UV-curable adhesive (NOA65) was dispensed around the active area using a dispenser system (SPEEDLINE-8000-1). The upper substrate with bare ITO was then aligned and laminated onto the lower substrate coated with PI and spacers. The bonding was completed using an assembly machine (SHINDO-ASSEMBLY M/C), which also cured the adhesive via UV exposure to maintain a consistent cell gap. LC (HBM-2) was introduced into the active area of the BD cell using a vacuum filling system (SHINDO-FILLING M/C) under a vacuum pressure of 5 × 10^−3^ Torr, with a filling time set to 180 min. Finally, end-sealing was performed using an end-seal machine (SHINDO-ENDSEAL M/C), where pressure was applied to the top and bottom plates, and the filling port was sealed using UV bonding. This completed the fabrication of the final BD cell.

### Experimental setup

All RGB diffraction angles of the BD were measured under an operating voltage of less than 10 V. The threshold for full phase modulation exceeding 2π at voltages below 10 V was confirmed using a Mach–Zehnder interferometer, validating effective LC-based phase control within this voltage range. A voltage range of 0–10 V was applied to the BD cell using an 8-bit gray scale (0–255 levels). For optical characterization, RGB laser beams with center wavelengths of 638 nm (R), 520 nm (G), and 460 nm (B) were individually directed onto the BD. A fiber-coupled laser system (OZ-RGB-Laser) with a maximum output power of 15 mW per channel was employed. The diffracted beams were captured and analyzed using a color CCD camera (model: BFS-U3-200S6C-C) to evaluate beam steering performance.

### Material properties

The LC mixture used in the BD was custom-synthesized by the coauthors. To enable minimal cell gap operation while ensuring sufficient phase retardation, a high-refractive-index nematic LC mixture (HBM-2) was developed and the material parameters of the HBM-2 nematic LC at room temperature are summarized as follows:

**Table:** 

Parameter	Quantity for HBM-2 Liquid Crystal
Dielectric Anisotropy (ε||)	16.1
Dielectric Anisotropy (ε⊥)	3.55
Ordinary refractive index *n*_o_ (*λ* = 460 nm/520 nm/638 nm)	1.5547/1.5428/1.5292
Extraordinary refractive index *n*_e_ (*λ* = 460 nm/520 nm/638 nm)	1.9198/1.8802/1.8333
Elastic Constant K_11_ (J/m)	26 × 10^−^^12^
Elastic Constant K_22_ (J/m)	5 × 10^−^^12^
Elastic Constant K_33_ (J/m)	29 × 10^−^^12^
Viscosity (Pa·s)	0.75

## Supplementary information

Supplementary Information

Supplementary Video

## Data Availability

The data supporting the findings of this study are available from the corresponding authors upon reasonable request.

## Code availability

The code used for this study is available from the corresponding author upon reasonable request.
