# Supplementary material for: Achromatic beam deflector with electrodynamic phased arrays
Source: Light Sci Appl. 2025 Aug 18;14:276. doi: 10.1038/s41377-025-01936-5 (PMC12358522; doi:10.1038/s41377-025-01936-5)
Supplement: Supplementary file 1 — Supplementary Information [file 41377_2025_1936_MOESM1_ESM.docx]

**Supplementary Information**

**Achromatic beam deflector with electrodynamic phased arrays**

**Jungkwuen An ^1, †^, Young Kim ^2, †^, Yunhee Kim ^1^, Hoon Song ^1^, Chunghwan Jung^3^, Kanghee Won ^4, *^, Junsuk Rho ^3, 5, 6, 7, *^, and Hong-Seok Lee ^8, *^**

^1^ Visual Technology Team, Samsung Research, Seoul 06765, Republic of Korea

^2^ Advanced Sensor Lab, Device Research Center, Samsung Advanced Institute of Technology, Suwon 16678, Republic of Korea

^3^ Department of Chemical Engineering, Pohang University of Science and Technology (POSTECH), Pohang 37673, Republic of Korea

^4^ Department of Information Display, Kyung Hee University, Seoul 02447, Republic of Korea

^5^ Department of Mechanical Engineering, Pohang University of Science and Technology (POSTECH), Pohang 37673, Republic of Korea

^6^ Department of Electrical Engineering, Pohang University of Science and Technology (POSTECH), Pohang 37673, Republic of Korea

^7^ POSCO-POSTECH-RIST Convergence Research Center for Flat Optics and Metaphotonics, Pohang 37673, Republic of Korea

^8^ Major of Electrical Engineering, College of Engineering, Pukyong National University, Busan 48513, Republic of Korea

* Corresponding authors: [khwon@khu.ac.kr](mailto:khwon@khu.ac.kr) (K.W.), [jsrho@postech.ac.kr](mailto:jsrho@postech.ac.kr) (J.R.), [lhs12100@pknu.ac.kr](mailto:lhs12100@pknu.ac.kr) (H.-S.L.)

† These authors contributed equally.

**Supplementary Note 1: The polarization states for the R, G, and B beams using Jones formalism**

The input polarization is horizontal polarized as follows,

$$\left. |H \right\rangle=\left( \begin{matrix} 1 \\ 0 \end{matrix} \right)$$

Jones matrix of retarder with arbitrary phase and azimuthal angle can be expressed as below.

$$\left. |H \right\rangle=e^{-\frac{i\eta}{2}}\left( \begin{matrix} \cos^{2} \theta+e^{i\eta}\sin^{2} \theta& \left( 1-e^{i\eta} \right)\cos\theta\sin\theta\\ \left( 1-e^{i\eta} \right)\cos\theta\sin\theta& \sin^{2} \theta+e^{i\eta}\cos^{2} \theta\end{matrix} \right)$$

where $\eta$ is the relative phase retardation induced between the fast axis and the slow axis and $\theta$ is the orientation of the fast axis with respect to the x-axis.

Thus, the Jones matrix of a color selective retarder at 22.5 degrees is written as following.

$J_{CSR22.5}=\frac{1}{\sqrt{2}}\left[ \begin{matrix} 1 & 1 \\ 1 & -1 \end{matrix} \right]$ for target wavelength

$J_{CSR22.5}=\left[ \begin{matrix} 1 & 0 \\ 0 & 1 \end{matrix} \right]$ for non-target wavelength

Jones matrix of a color selective retarder at 45 degrees is written as following.

$J_{CSR45}=\left[ \begin{matrix} 0 & 1 \\ 1 & 0 \end{matrix} \right]$ for target wavelength

$J_{CSR45}=\left[ \begin{matrix} 1 & 0 \\ 0 & 1 \end{matrix} \right]$ for non-target wavelength

Jones matrix of a half-waveplate at 45 degrees is written as following.

$J_{HWP45}=\left[ \begin{matrix} 0 & 1 \\ 1 & 0 \end{matrix} \right]$

Jones matrix of a vertical beam deflector is written as following.

$J_{BDV}=\left[ \begin{matrix} 0 & 0 \\ 0 & 1 \end{matrix} \right]$ for the deflected light

$J_{BDV}=\left[ \begin{matrix} 1 & 0 \\ 0 & 0 \end{matrix} \right]$ for the transmitted light

Jones matrix of a horizontal beam deflector is written as following.

$J_{BDH}=\left[ \begin{matrix} 1 & 0 \\ 0 & 0 \end{matrix} \right]$ for the deflected light

$J_{BDH}=\left[ \begin{matrix} 0 & 0 \\ 0 & 1 \end{matrix} \right]$ for the transmitted light

Jones matrix of a horizontal polarizer is written as following.

$J_{POL}=\left[ \begin{matrix} 1 & 0 \\ 0 & 0 \end{matrix} \right]$

Then the Jones matrix of each color deflector module can be written as follows.

$$J=J_{POL}\cdot J_{CSR22.5}\cdot J_{BDV}\cdot J_{HWP45}\cdot J_{BDH}\cdot J_{CSR45}\cdot J_{BDH}\cdot J_{HWP45}\cdot J_{BDV}\cdot J_{CSR22.5}$$

For example, the polarization evolution at the green channel #1 at the green deflector module can be calculated as follows.

$$\left. |\psi_{G\#1} \right\rangle=J_{G}\cdot\left. |H \right\rangle$$

$$=\left[ \begin{matrix} 1 & 0 \\ 0 & 0 \end{matrix} \right]\cdot\frac{1}{\sqrt{2}}\left[ \begin{matrix} 1 & 1 \\ 1 & -1 \end{matrix} \right]\cdot\left[ \begin{matrix} 1 & 0 \\ 0 & 0 \end{matrix} \right]\cdot\left[ \begin{matrix} 0 & 1 \\ 1 & 0 \end{matrix} \right]\cdot\left[ \begin{matrix} 0 & 0 \\ 0 & 1 \end{matrix} \right]\cdot\left[ \begin{matrix} 0 & 1 \\ 1 & 0 \end{matrix} \right]\cdot\left[ \begin{matrix} 1 & 0 \\ 0 & 0 \end{matrix} \right]\cdot\left[ \begin{matrix} 0 & 1 \\ 1 & 0 \end{matrix} \right]\cdot\left[ \begin{matrix} 0 & 0 \\ 0 & 1 \end{matrix} \right]\cdot\frac{1}{\sqrt{2}}\left[ \begin{matrix} 1 & 1 \\ 1 & -1 \end{matrix} \right]\cdot\left( \begin{matrix} 1 \\ 0 \end{matrix} \right)$$

$$=\frac{1}{2}\left( \begin{matrix} 1 \\ 0 \end{matrix} \right)$$

In the other hand, the polarization evolution at the green channel #2 at the green deflector module can be calculated as follows.

$$\left. |\psi_{G\#2} \right\rangle=J_{G}\cdot\left. |H \right\rangle$$

$$=\left[ \begin{matrix} 1 & 0 \\ 0 & 0 \end{matrix} \right]\cdot\frac{1}{\sqrt{2}}\left[ \begin{matrix} 1 & 1 \\ 1 & -1 \end{matrix} \right]\cdot\left[ \begin{matrix} 0 & 0 \\ 0 & 1 \end{matrix} \right]\cdot\left[ \begin{matrix} 0 & 1 \\ 1 & 0 \end{matrix} \right]\cdot\left[ \begin{matrix} 1 & 0 \\ 0 & 0 \end{matrix} \right]\cdot\left[ \begin{matrix} 0 & 1 \\ 1 & 0 \end{matrix} \right]\cdot\left[ \begin{matrix} 0 & 0 \\ 0 & 1 \end{matrix} \right]\cdot\left[ \begin{matrix} 0 & 1 \\ 1 & 0 \end{matrix} \right]\cdot\left[ \begin{matrix} 1 & 0 \\ 0 & 0 \end{matrix} \right]\cdot\frac{1}{\sqrt{2}}\left[ \begin{matrix} 1 & 1 \\ 1 & -1 \end{matrix} \right]\cdot\left( \begin{matrix} 1 \\ 0 \end{matrix} \right)$$

$$=\frac{1}{2}\left( \begin{matrix} 1 \\ 0 \end{matrix} \right)$$

As you have seen, the polarizer at the end of the module reduces the total power by half, since the power is $P=\left\langle\psi| \psi^{*} \right\rangle=\left( \begin{matrix} 1/2 & 0 \end{matrix} \right)\left( \begin{matrix} 1/2 \\ 0 \end{matrix} \right)=\frac{1}{4}$. The channel #1 has a quarter of the input power and the channel #2 has the same as the channel #1. Meanwhile, the beam with green wavelength at the other deflector module, such as a red deflector can be written as follows,

$$\left. |\psi_{G} \right\rangle=J_{R}\cdot\left. |H \right\rangle$$

$$=\left[ \begin{matrix} 1 & 0 \\ 0 & 0 \end{matrix} \right]\cdot\left[ \begin{matrix} 1 & 0 \\ 0 & 1 \end{matrix} \right]\cdot\left[ \begin{matrix} 1 & 0 \\ 0 & 0 \end{matrix} \right]\cdot\left[ \begin{matrix} 0 & 1 \\ 1 & 0 \end{matrix} \right]\cdot\left[ \begin{matrix} 0 & 0 \\ 0 & 1 \end{matrix} \right]\cdot\left[ \begin{matrix} 1 & 0 \\ 0 & 1 \end{matrix} \right]\cdot\left[ \begin{matrix} 0 & 0 \\ 0 & 1 \end{matrix} \right]\cdot\left[ \begin{matrix} 0 & 1 \\ 1 & 0 \end{matrix} \right]\cdot\left[ \begin{matrix} 1 & 0 \\ 0 & 0 \end{matrix} \right]\cdot\left[ \begin{matrix} 1 & 0 \\ 0 & 1 \end{matrix} \right]\cdot\left( \begin{matrix} 1 \\ 0 \end{matrix} \right)$$

$$=\left( \begin{matrix} 1 \\ 0 \end{matrix} \right)$$

It means that the field is conserved, so there is no power losses during the polarization manipulation, when the wavelength is not the target wavelength of the deflector module. Then the final polarization state of green light out of the dual-channel achromatic beam deflector can be written as follows.

$$\left. |\psi_{G\#1} \right\rangle=J_{B}\cdot J_{G}\cdot J_{R}\cdot\left. |H \right\rangle=\left( \begin{matrix} 1/2 \\ 0 \end{matrix} \right)$$

$$\left. |\psi_{G\#2} \right\rangle=J_{B}\cdot J_{G}\cdot J_{R}\cdot\left. |H \right\rangle=\left( \begin{matrix} 1/2 \\ 0 \end{matrix} \right)$$

It applies for both red and blue wavelength as well. In the case of single-channel achromatic deflector module, there is no power losses due to the polarization manipulation.

**Supplementary Note 2: Specific data on intensity loss, including insertion loss and depolarization.**

The intensity of incident light is quantitatively measured based on the output voltage (*V*_out_) generated by the photodiode's current-to-voltage (I-V) conversion circuit. This conversion follows the fundamental relationship where the photocurrent (*I*_ph_​) is directly proportional to the optical power (*P*_opt_) received by the photodiode, as expressed by:

*I_ph_=R_λ_⋅P_opt_*

where *R*_λ_ represents the responsivity of the photodiode (A/W) at a specific wavelength. The generated photocurrent is subsequently converted into a measurable voltage using a transimpedance amplifier (TIA) with a feedback resistor (*R*_f_), yielding the output voltage:

*V_out_=I_ph_⋅R_f_*

Thus, the output voltage serves as a direct indicator of light intensity, modulated by the photodiode’s responsivity and the amplification factor determined by *R_f_*. The optimal gain value (G) is carefully adjusted to ensure precise signal acquisition, maximizing signal-to-noise ratio (SNR) while maintaining the necessary bandwidth for the application. The optimized gain values and corresponding measurement parameters are systematically presented in the table below.

| **Component** | **Voltage (V)** | **Efficiency (%)** | **Loss (%)** |
| --- | --- | --- | --- |
| Incident Light | 2.76 | - | - |
| CSR 22.5° | 2.76 | 100.0 | 0.0 |
| HWP | 2.47 | 89.4 | 10.6 |
| BD | 2.27 | 82.2 | 17.8 |
| CSR 45° | 2.68 | 97.1 | 2.9 |
| Linear Polarizer | 2.45 | 88.8 | 11.2 |

The overall efficiency for the unit BD for red in Fig. 3 can be expressed as follows.

CSR 22.5° (1) * BD (0.82) * HWP 45 (0.89) * BD (0.82) * CSR 45° (0.97) * BD (0.82) * HWP 45 (0.89) * BD (0.82) * CSR 22.5 (1) * LP (0.89) = 0.309.

It is attributed to depolarization occurring within the LC layer, the retardation films, and reflections at the glass surface. Depolarization can partially result from phase differences between polarization components caused by the misalignment of LC molecules due to irregularities in the rubbing process. Additionally, it can also be induced by light scattering from spacer balls used to maintain the uniform thickness of the LC cell. This can be accomplished by minimizing alignment defects through methods such as precise rubbing techniques or photoalignment, utilizing high-purity LC materials, and employing optimized anchoring layers to mitigate depolarization effects. Additionally, applying anti-reflection coatings and index-matching layers can help reduce multiple reflections and the associated polarization changes.

**Supplementary Note 3: Maximum diffraction efficiency of the ideal beam deflector with 2 micron pitch for each R/G/B wavelengths**

**(a)**


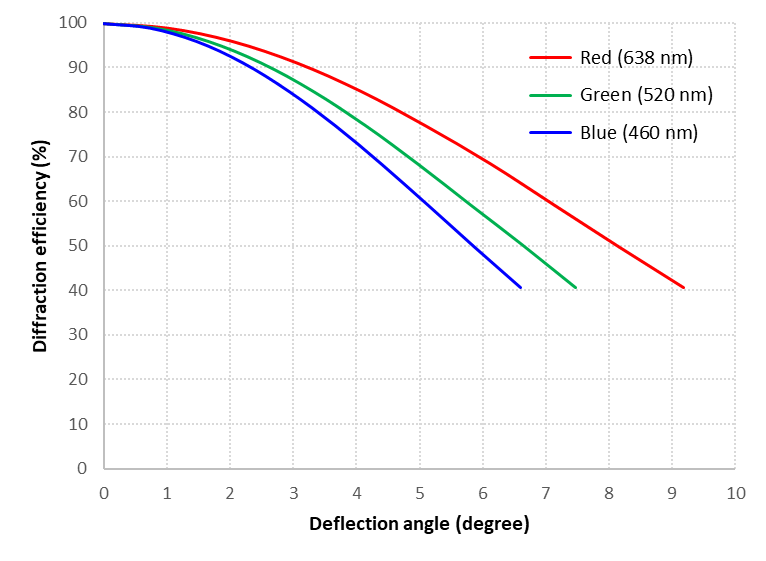


**(b) (c) (d)**


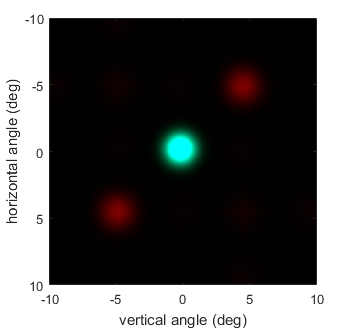

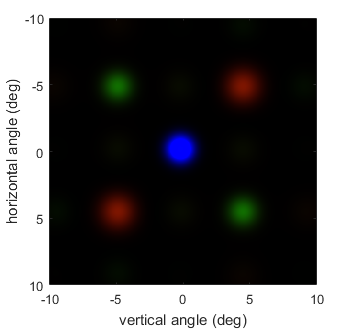

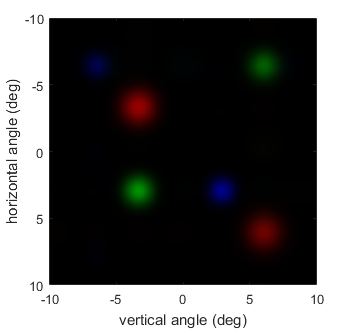


Fourier optic simulation of (a) Maximum diffraction efficiency of the ideal beam deflector with 2 micron pitch for R, G, B wavelength. (b) Only red incident light is deflected into two channels while green and blue incident lights still remain in DC without deflecting, (c) Only green incident light deflected into two channels following the previously deflected red incident light. The blue incident light does not deflect and still remains in DC, (d) blue light is deflected into two channels.

**Supplementary Note 4: Adapting Electrodynamic Phased Arrays for mmWave Frequencies**

mmWave technology plays a pivotal role across various industries, demonstrating its versatility and potential. In telecommunications, it serves as a cornerstone for 5G and 6G networks by enabling ultra-fast data transmission and low-latency communication. Its precision and high-resolution capabilities make it indispensable in sensing applications, such as autonomous vehicle radars, airport security scanners, and advanced imaging systems.

Electrodynamic phased arrays are a promising technology for beam steering devices in the mmWave frequency range, playing a critical role in sensors and 5G/6G technologies.

In sensors, these devices are integral to autonomous driving radars, providing 360° detection, high-resolution tracking, and precise distance and speed measurements. They are also used in security and imaging systems, enabling non-contact scanning and precise differentiation of materials in applications like airport security and drone-mounted sensors.

In telecommunications, beam steering enhances 5G/6G networks by focusing signals toward users, dynamically tracking mobile devices, reducing interference, and improving energy efficiency. In satellite communications, it ensures stable data links, enables multi-user connections, and provides global coverage with ultra-low latency. For smartphones and IoT devices, beam steering improves signal quality, enhances connectivity, and supports smart home and autonomous drone operations.

Adapting electrodynamic phased arrays can also be applied to circularly polarized light (CPL). Efficient CPL control minimizes light loss and ensures uniform optical signal transmission. Specifically, by leveraging the birefringent properties of LCs, linearly polarized light can be transformed into CPL based on the molecular alignment of the LC layer. This allows for optimized phase retardation, maximizing CPL conversion efficiency at a targeted wavelength. Additionally, integrating metasurfaces with LCs enables selective filtering or conversion of CPL in a specific direction. LC-based lenses and beam steering devices exhibit strong potential for phase modulation and beam steering in the mmWave frequency range, as well as precise control of CPL.

However, challenges such as significant signal loss, high power consumption, and heat generation at mmWave frequencies require careful design, material losses, and efficient thermal management solutions.

Future research should focus on advanced materials like graphene, perovskites, liquid crystals, multi-band operation, and AI-driven optimization to enhance functionality and adaptability. These efforts will address existing challenges and unlock the full potential of electrodynamic phased arrays in next-generation communication technologies.
